# Reconstruction of Nickel Chalcogenide Induced Ruthenium Nanoparticles Embedding for Oxygen Evolution: Mechanism Switching Enables Enhanced Catalytic Activity

**DOI:** 10.1002/advs.76026

**Published:** 2026-06-09

**Authors:** Yuewen Wu, Mingpeng Chen, Xinqi Chen, Huachuan Sun, Tong Zhou, Yun Chen, Dequan Li, Jin Zhang, Feng Liu, Hao Cui, Qingju Liu

**Affiliations:** ^1^ Yunnan Key Laboratory for Micro/Nano Materials & Technology National Center for International Research on Photoelectric and Energy Materials School of Materials and Energy Yunnan University Kunming China; ^2^ Yunnan Precious Metals Laboratory Co., Ltd. Kunming China

**Keywords:** mechanism switching, metal‐support catalyst, reconstruction, Ru embedding, water oxidation

## Abstract

Atomic‐level understanding of structure‐performance relationships and electrocatalytic mechanisms is pivotal for efficient oxygen evolution reaction (OER). Unlike conventional strategies that focus on developing pre‐catalysts, a unique design conception is proposed in this work to maximize metal‐support interactions through reconstruction. We prepare the ruthenium nanoparticles supported nickel chalcogens (Ru/NiX, X = S, Se), which enable fast electrooxidation to form an adaptively Ru embedding structure, featured by the compressed Ru─O─Ni bridge bonds at the Ru/NiOOH interface. Combined experimental analyses and theoretical calculations reveal that both thermodynamics and kinetics of the OER process are optimized. On the one hand, Ru nanoparticles have a high affinity for capturing the OH^−^, consequently increasing the *O radical coverage. On the other hand, a shorter inter atomic Ru─Ni distance facilitates the *O–*O radical coupling, validating the mechanism switching from the adsorbate evolution mechanism (AEM) to the oxide path mechanism (OPM). The reconstructed catalyst merely requires an overpotential of 175 mV at 10 mA cm^−2^ and maintains stable operation for over 250 h, implying a superior OER performance.

## Graphical abstract

Ru nanoparticles supported nickel chalcogens (Ru/NiX, X = S, Se), which enable fast electrooxidation to form an adaptively Ru embedding structure, featured by the compressed Ru─O─Ni bridge bonds at the Ru/NiOOH interface. On the one hand, Ru nanoparticles have a high affinity for capturing the OH^−^, consequently increasing the *O radical coverage. On the other hand, a shorter inter atomic Ru─Ni distance facilitates the *O–*O radical coupling, validating the mechanism switching from the adsorbate evolution mechanism (AEM) to the oxide path mechanism (OPM).

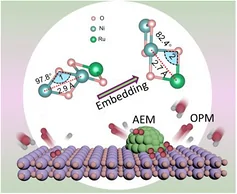

## 1 Introduction

The oxygen evolution react limitations as a crucial anodic half‐reaction, provides the essential protons and electrons through water oxidation that are required to drive the cathodic processes, such as CO_2_/N_2_ reduction or fuel generation [1, 2, 3]. However, the sluggish kinetics of OER at the anode remains a critical bottleneck limiting the overall efficiency [4, 5, 6]. OER generally involves four proton‐coupled electron transfer steps under harsh oxidative and acidic/alkaline environments, calling for high standards to catalyst's activity and durability [7, 8, 9]. Although noble‐metal‐based catalysts such as IrO*_x_* or RuO*_x_* exhibit outstanding intrinsic activity, their scarcity, high cost, and poor stability hinder large‐scale deployment [10, 11, 12]. To overcome these limitations, intensive efforts have been devoted to exploiting 3d transition‐metal‐based catalysts and multicomponent catalysts [13, 14, 15].

Extensive studies on state‐of‐the‐art catalysts have revealed that the alkaline OER generally proceeds via three distinct mechanisms: the adsorbate evolution mechanism (AEM), the lattice oxygen mechanism (LOM), and the oxide pathway mechanism (OPM) [16, 17, 18]. The AEM occurs between active sites and oxygen intermediates (*OH, *O, *OOH, and O_2_), limiting the improvement of catalytic activity through linear scaling relationships in the adsorption energy of these intermediates [19, 20, 21]. Similarly, LOM proceeds via the involvement of lattice oxygen to directly generate *OH, $O_{2}^{2-}$, and O_2_, leading to severe dissolution of active sites [22, 23, 24]. In contrast, the OPM directly binds adjacent *OH species for *O–*O coupling to produce O_2_, without generating oxygen vacancies and *OOH intermediates [25, 26, 27]. Thus, OPM is able to break through the bottleneck poor stability in LOM and finite activity in AEM.

To tailor a catalyst that customizes the OPM mechanism, it is vital to design appropriate atomic spacing between metal sites [28, 29, 30]. Consequently, the adsorption of *OH species on dual sites can facilitate the direct *O–*O radical coupling. With the methods of heteroatom doping, lattice strain, and vacancy engineering, the atomic spacing can be precisely shortened and M–O hybridization can be enhanced, thereby accelerating the kinetics of *OH adsorption and deprotonation [31, 32]. For example, in heterogeneous RuO_2_–CeO_2_ electrocatalysts, interface and non‐interface RuO_2_ sites selectively activate OPM and enhanced AEM, respectively. The Ru─O─Ce bridge at the interface facilitates electron transfer from Ru to Ce, thus the adjacent Ru and Ce sites at this interface initiate the OPM. The catalyst exhibits an extremely low overpotential of 180 mV at 10 mA cm^−2^ [33]. For another example, the high coverage of the Pt interface on the RuO_2_‐Pt catalyst contributes up to 48% to the *O coupling step (*O–*O formation). The OER reaction dominated by the OPM mechanism reduces the required potential at a current density of 10 mA cm^−2^ from ∼300 to ∼200 mV [34, 35]. Metal‐support regulations can not only regulate the electronic structures of active sites, but also effectively switch the OER mechanism in a customized manner [36, 37, 38, 39].

Herein, a surface‐embedding strategy is proposed to prepare Ru/NiOOH through anchoring Ru nanoparticles on the surface of nickel chalcogenides (NiX) and activating surface reconstruction in the OER regions [40]. Compared to the non‐interface Ni sites, the interfacial Ru─O─Ni bonds greatly shorten the distance between Ru and Ni atoms. In situ characterization and theoretical calculations reveal that adaptive surface reconstruction embedding drives a mechanistic transition from the AEM to the OPM. The mechanism switching has structurally occurred by a compressed dual‐site distance of Ru−Ni (2.7 Å) and moderate intermediate *OH/*O adsorption energies, which significantly lowers the energy barrier of direct *O–*O coupling and expedites OER kinetics. Thus, Ru/NiX demonstrates extraordinary OER performance with an ultralow overpotential (< 180 mV) and satisfactory stability.

## 2 Results

### 2.1 Structural Characterizations and Theoretical Validation

**FIGURE 1:**
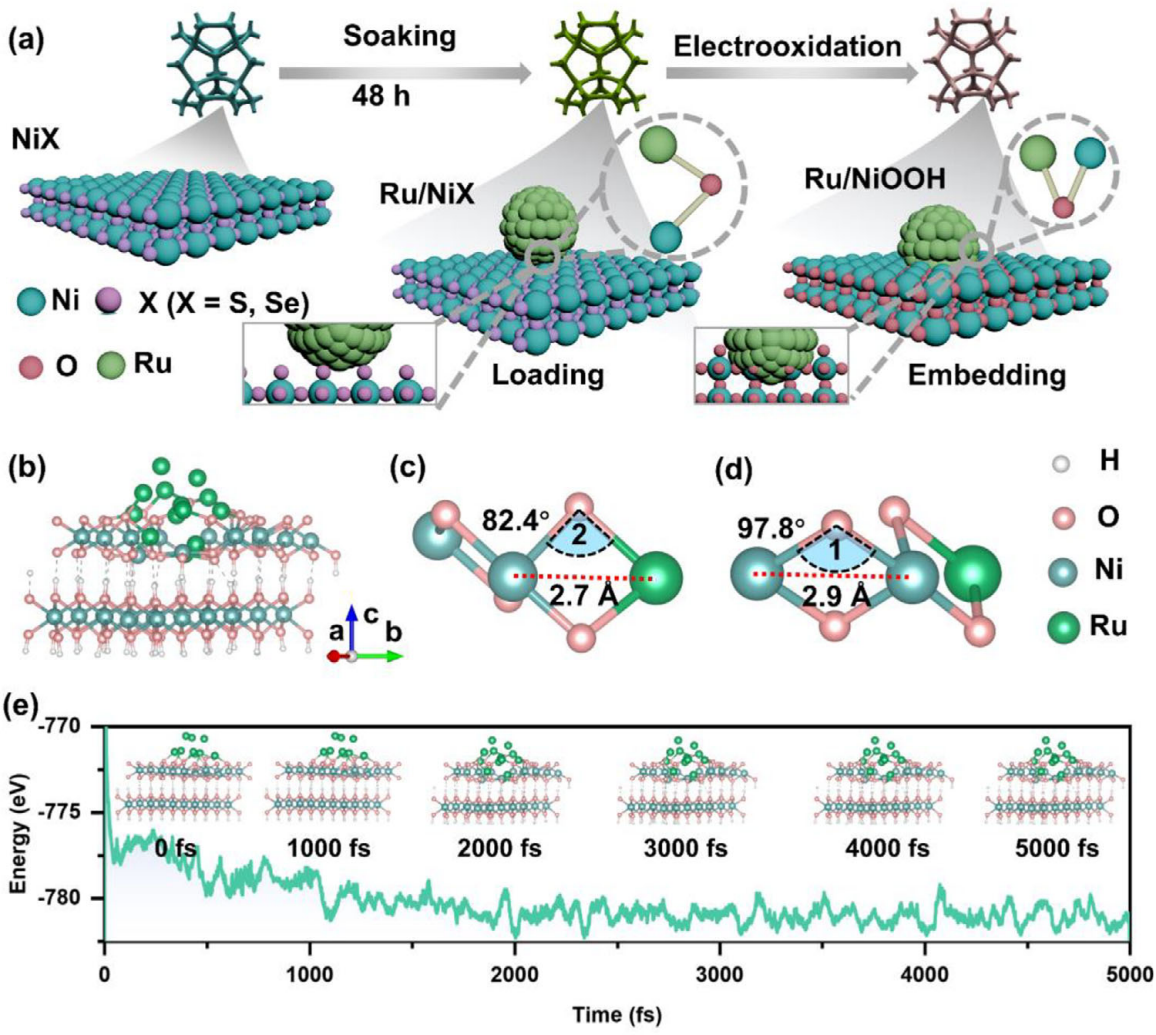
Preparation and theoretical validation. (a) Scheme of the synthetic processes of Ru/NiOOH through Ru nanoparticle embedding and electrochemical activation. (b) Simulated structure after electrooxidation. (c, d) Theoretical models of reconstructed Ru─O─Ni and Ni─O─Ni configurations. (e) AIMD simulation for Ru/NiOOH under OER conditions.

NiX (X = S, Se) was synthesized on NF substrate using a hydrothermal method, and Ru/NiX pre‐catalyst was obtained by soaking in Ru^3+^ solution at room temperature. Ru nanoparticles are embedded into NiOOH through the in situ surface reconstruction (Figure 1a). This process leads to extra lattice compression and deformation between Ru particles and NiOOH.

The theoretical model of Ru/NiOOH is shown in Figure 1b, and the Ru─O─Ni bond angle at the interface is calculated to be 82.4°, while the Ni─O─Ni bond angles at non‐interface positions are estimated to be 97.8° (Figure 1c,d). As a result, the distance between Ru─Ni is 2.7 Å, while that of Ni─Ni is 2.9 Å. With the compression of Ru─O─Ni unit, strong metal‐support interaction occurs to interfere with the possible reaction pathway [39]. To verify the feasibility of this structure, ab initio molecular dynamics (AIMD) simulations were conducted to evaluate the thermal stability of Ru/NiOOH, which remains intact for 5000 fs under T = 300 K. As shown in Figure 1e, the total energy of Ru/NiOOH continues to decrease from −777 to −781 eV at the first 2000 fs, which accompanies Ru nanoparticles sinking into the NiOOH subsurface. In the range of 2000–5000 fs, the energy fluctuates around the equilibrium value of −781 eV, and the whole structure shows rare change, elucidating its excellent stability in operation.

**FIGURE 2:**
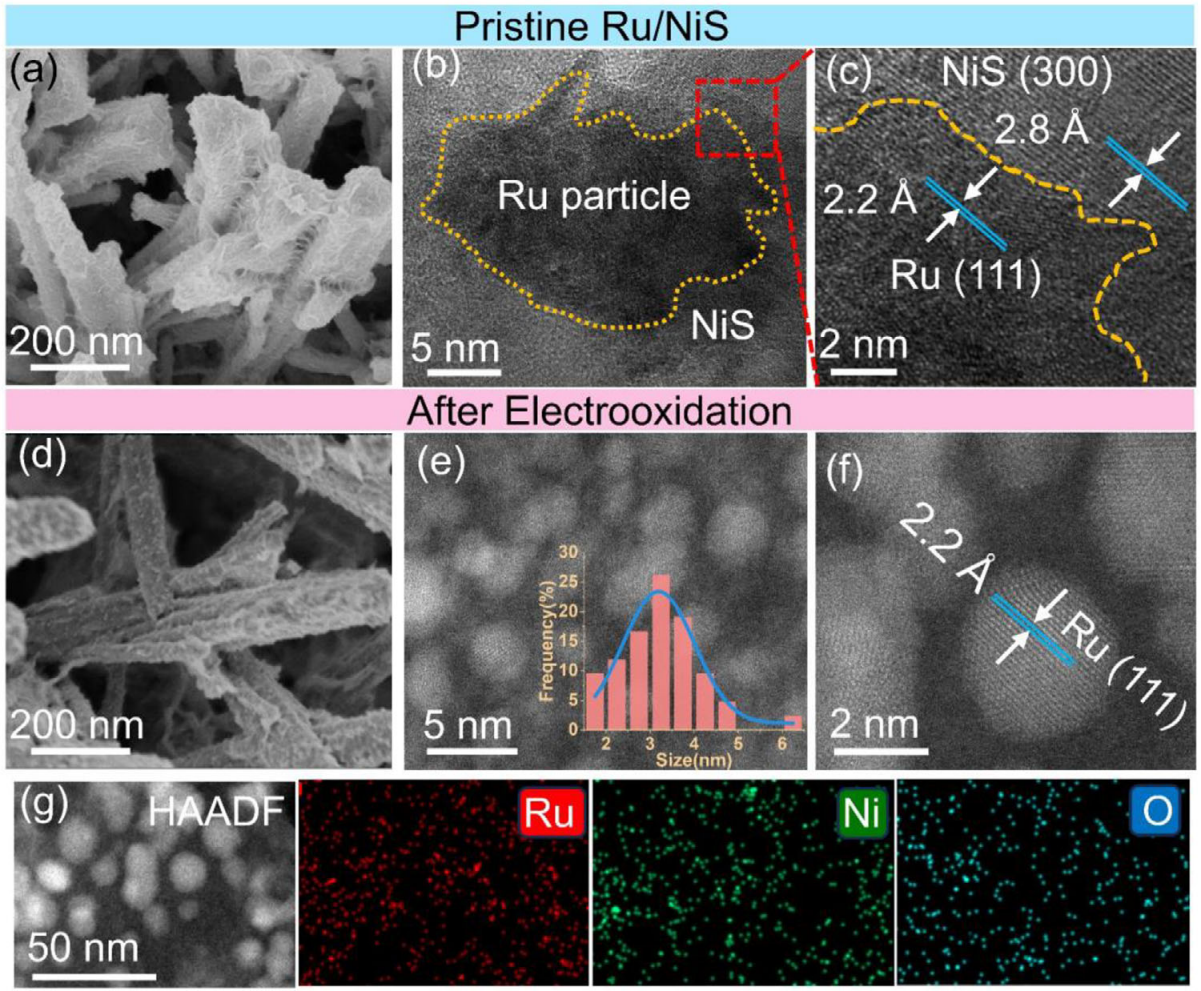
Structural characterizations. (a–c) SEM, TEM, HRTEM image of Ru/NiS. (d) SEM, (e, f) HAADF‐STEM image, and (g) EDS mapping of Ru/NiOOH.

The compositional and structural information of the synthesized pre‐catalyst was acquired by using X‐ray diffraction (XRD). As shown in Figure S1, the characteristic peaks at 18.5°, 32.3°, 37.4°, 40.5°, and 49.0° are indexed to NiS (PDF #86‐2280). The geometric morphology was analyzed by scanning electron microscope (SEM). The NiS precursor demonstrates connected and uniform nanorods structure, and each nanorod shows an average diameter of ∼160 nm and a long length behind 1 µm (Figure S2a,b). As shown in Figure 2a and Figure S3a,b, after Ru species cover the surface of NiS nanorods, the smooth surface turns rough and hierarchical. The fine structure of crystalline Ru/NiS catalyst was studied by using transmission electron microscopy (TEM) and high‐resolution TEM (HRTEM). In Figure S4a,b, the size of Ru/NiS hierarchical nanorod shows good consistency with the SEM image. The HRTEM image (Figure 2b) reveals that an irregular Ru particle is loaded on NiS. As shown in Figure 2c and Figure S4c, the interplanar spacing of 2.2 Å belongs to the (111) crystal plane of Ru metal, while the interplanar spacing of 2.8 Å corresponds to the (300) crystal plane of NiS. The selected electron diffraction pattern (Figure S4d) is consistent with the HRTEM and XRD results, suggesting distinct characteristic signals of Ru and NiS crystals. After electrooxidation, the nanorods remain intact, but their surface is covered with a granular texture (Figure 2d). The magnified high‐angle annular dark field scanning transmission electron microscope (HAADF‐STEM) image confirms the homogeneous distribution of Ru spherical particles. Figure 2e indicates that the average size of Ru particles is about ∼3.2 nm. Figure 2f again confirms that Ru exposes the (111) crystal plane with a lattice spacing of 2.2 Å. The above evidence indicates the existence of Ru nanoparticles [41].

### 2.2 Electronic and Coordination Structures

**FIGURE 3:**
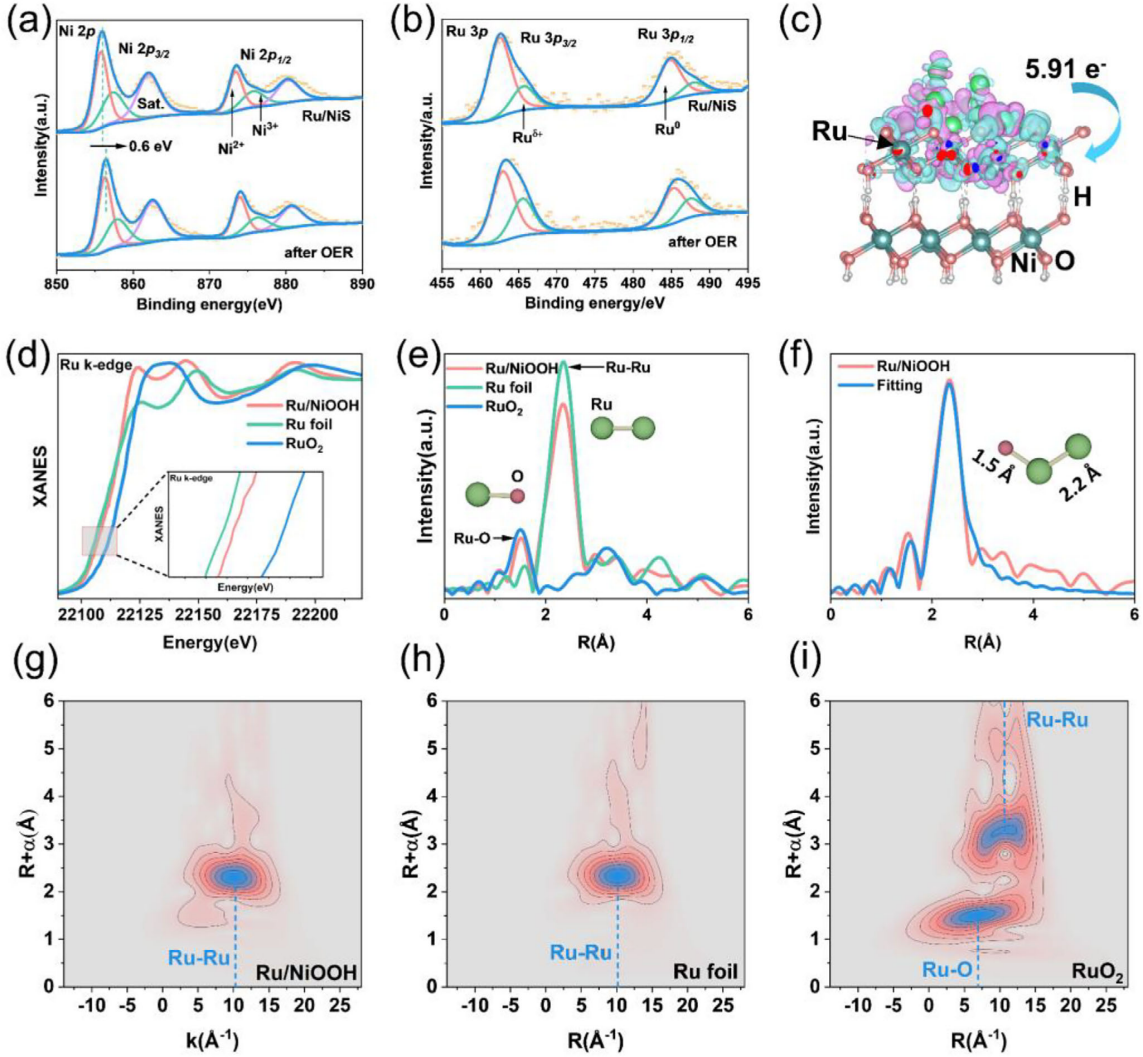
Electronic and coordination structures. (a) Ni 2p, (b) Ru 3p high‐resolution XPS spectra of Ru/NiS and Ru/NiOOH. (c) Charge density difference maps of Ru/NiOOH. (d) Ru *K*‐edge X‐ray absorption near‐edge structure (XANES) spectra for Ru/NiOOH, RuO_2_, and Ru foil. (e) Fourier transform of Ru *K*‐edge EXAFS spectra. (f) Fitting result. (g–i) Wavelet transform for the *k* ^3^‐weight EXAFS of Ru/NiOOH, Ru foil, and RuO_2_.

Afterward, X‐ray photoelectron spectroscopy (XPS) tests were conducted on Ru/NiS and Ru/NiOOH to investigate the changes in electronic structures. Figure 3a shows that a deconvolved peak at the binding energy of 856.1 eV in Ni 2p_3/2_ can be assigned to Ni^2+^ for Ru/NiS. After electrooxidation, the peak positively shifts by 0.6 eV, closer to the Ni^3+^ state, indicating the appearance of NiOOH on the surface [42]. In addition, the peak at 462.6 eV in the Ru 3p_3/2_ spectra (Figure 3b) is attributed to Ru^0^, which exists in both Ru/NiS and Ru/NiOOH, indicating the stable existence of Ru nanoparticles. The deconvoluted peak of Ru 3p_3/2_ with a binding energy of 466.0 eV is considered as Ru^δ+^. The area of Ru^δ+^ significantly increases after electrooxidation, indicating an increase in the number of high‐valence Ru species. This is due to the embedding of Ru particles into the surface of NiOOH after reconstruction, leading to the formation of more Ru─O pockets, further confirming the embedding behavior of Ru particles [43]. The XPS spectrum of O 1s is shown in Figure S5. The peaks at binding energies of 531.3 and 532.4 eV are attributed to adsorbed oxygen and oxygen in water. Notably, no lattice oxygen peak appears near the binding energy of 530.0 eV, providing preliminary evidence that the LOM mechanism does not exist in the catalyst. The binding energy shift of Ru/NiSe before and after the OER is almost identical to that of Ru/NiS (Figure S6a,b). To gain a deeper understanding of the electronic interaction between metal and carrier, we simulated the stable state model of Ru embedded in NiOOH as shown in Figure 3c, using DFT calculations [44]. The charge density difference calculation result indicates that the total Ru species (13 Ru atoms) in the theoretical model transfers 5.91 e^−^ to the NiOOH, with an average electron loss of 0.46 e^−^ for Ru atoms, proving a strong metal‐support interaction in the Ru/NiOOH structure (Table S1).

To further explore the chemical state and local coordination environment of Ru in the post OER sample, X‐ray absorption spectroscopy data were collected. As shown in Figure 3d, the Ru absorption edge energy of Ru/NiOOH appears around 22 105.0 eV, which is lower than that of RuO_2_ (22 111.0 eV) and closer to that of Ru foil (22 103.5 eV) [45, 46, 47, 48]. Thus, the average oxidation state δ of Ru is between Ru foil (0) and RuO_2_ (+4), and the fitting result indicates an average valence of +0.9. This result is consistent with the XPS results, proving the co‐existence of zero‐valence Ru particles and low‐valence Ru^+δ^ at the metal‐support interface. The extended edge XAFS (EXAFS) results shown in Figure 3e indicate the presence of significant Ru‐Ru (2.2 Å) and weak Ru─O bonds (1.5 Å) in Ru/NiOOH, respectively, originating from the Ru─O─Ni bridge bonds between the Ru particles and the metal support contact [33]. The above parameters are based on the good‐fitting results provided in Figure 3f and Table S2. The wavelet transform with *k* ^3^ weights (Figure S7) was applied to EXAFS, and the results are shown in Figure 3g–i. The Ru─Ru bond and Ru─O bond with the *R*‐space once again confirm the existence of Ru nanoparticles.

### 2.3 Electrocatalytic Performance of Catalysts

**FIGURE 4:**
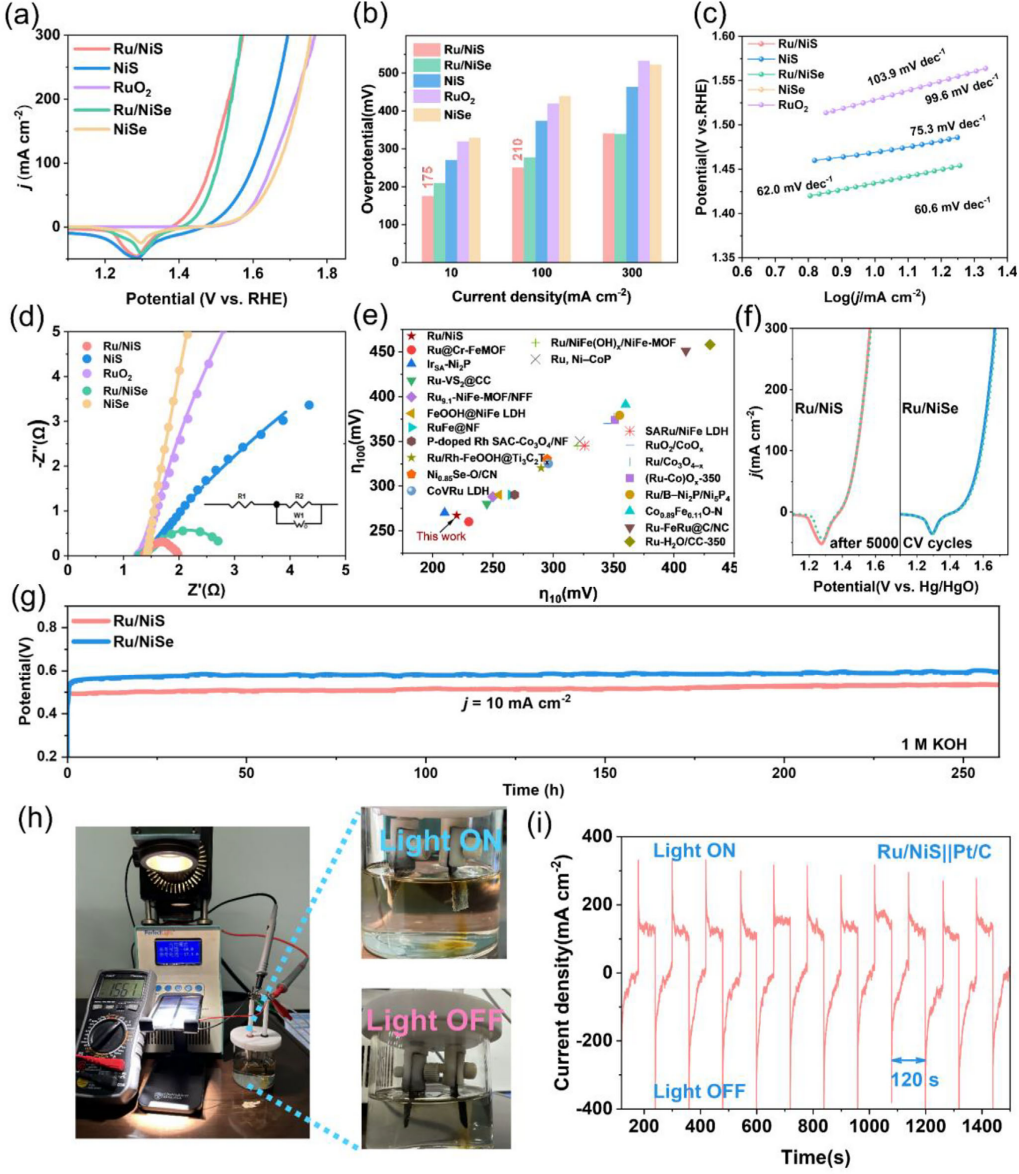
Electrocatalytic performance. (a) Polarization curves, (b) Overpotential, (c) Tafel plots, and (d) EIS curves of the prepared catalyst. (e) Activity comparison at 10 and 100 mA cm^−2^ between Ru/NiX and reported representative OER electrocatalysts. (f) LSV curves of Ru/NiX before and after 5000 CV cycles. (g) Long‐term stability test. (h) Actual operation photo of water splitting powered by a solar cell. (i) The start‐stop current‐time curve of the photovoltaic‐driven system.

To reveal the structure‐performance relationships, Figure 4a shows the linear sweep voltammetry (LSV) curve of the prepared electrode in 1 m KOH. Optimized Ru/NiS and Ru/NiSe exhibit extremely low overpotentials, requiring only 175 and 210 mV to achieve a current density of 10 mA cm^−2^ (Figure S8). Besides, NiS, NiSe, RuO_2_ electrodes (Figure 4b) exhibit inferior activity. To verify the consistency of the results, Figure 4c presents the Tafel slope curve for comparing the activity of the catalyst. Compared with the original NiX, the presence of Ru greatly optimized the performance, with the Tafel slope of Ru/NiX reduced to 60.6 and 62.0 mV dec^−1^, much lower than NiS (75.3 mV dec^−1^), RuO_2_ (99.6 mV dec^−1^), and NiSe (103.9 mV dec^−1^), indicating that Ru particles significantly enhance the catalytic activity of NiX. For objective comparison, Ru/NiX and other prepared catalysts were compared by normalizing LSV curves using double‐layer capacitance (C_dl_, Figure S9a–d), Ru/NiS exhibits excellent C_dl_ and normalized curves under 38.0 mF cm^−2^ (Figure S10). Figure 4d provides relevant electrochemical impedance spectroscopy (EIS) to demonstrate the correlation between conductivity and catalytic activity. The charge transfer resistance (R_ct_) of Ru/NiS and Ru/NiSe in Table S3 has been optimized to 0.9 and 1.8 Ω, further indicating that the reaction pathway followed by Ru/NiX can not only accelerate carrier migration, but also provide higher reaction activity. Furthermore, the O_2_ amount collected experimentally matches well with the theoretical value, leading to a high Faradaic efficiency of 98.3% of Ru/NiS for OER (Figure S11). At low overpotentials of 10 and 100 mA cm^−2^, the OER activity of Ru/NiX also exceeded many recently reported OER electrocatalysts, as shown in Figure 4e and Table S4. In addition, Ru/NX exhibits excellent electrochemical stability, with almost no performance degradation observed from the LSV curve after 5000 CV cycles, as shown in Figure 4f. When Ru/NiX is used as an anode, it can operate continuously for over 250 h with almost no attenuation at a current density of 10 mA cm^−2^ (Figure 4g). Additionally, the anode also remains stable at a higher current density of 250 mA cm^−2^ for 240 h (Figure S12). Table S5 shows the ICP results, and the dissolved amount of Ru and Ni during the OER process is 568.6 and 323.1 ug/L, indicating that Ru and Ni dissolve less during the electrochemical process. SEM and TEM images (Figure S13a–f) well indicate that the catalyst maintains the core–shell nanorods structure after long‐term OER stability. The HAADF‐STEM image (Figure S13g,h) indicates the stable existence of Ru particles and the uniform distribution of Ru, Ni, and O elements (Figure S13i).

Afterward, the electrode was assembled in an electrolysis device to prove its potential applications in green energy‐to‐hydrogen systems. As shown in Figure 4h, a xenon lamp was used to simulate a controllable solar driven Ru/NiS||Pt/C (20 wt.%) hydrogen production device, with silicon solar panels as the power source. Under simulated sunlight, H_2_ and O_2_ are continuously generated on the cathode and anode sides at a voltage of ∼1.5 V. At the same time, the lifespan loss of the catalyst is tested through response experiments controlled by light on/off signals. Figure 4i indicates that no bubbles or currents were observed on the electrode surface when the lamp was turned off. For several lamp on/off cycles with intervals of 60 s, the catalytic system exhibited good response and almost no attenuation, indicating the reliability of the solar driven Ru/NiX catalytic hydrogen production system.

### 2.4 OER Kinetics and Dual Pathways

**FIGURE 5:**
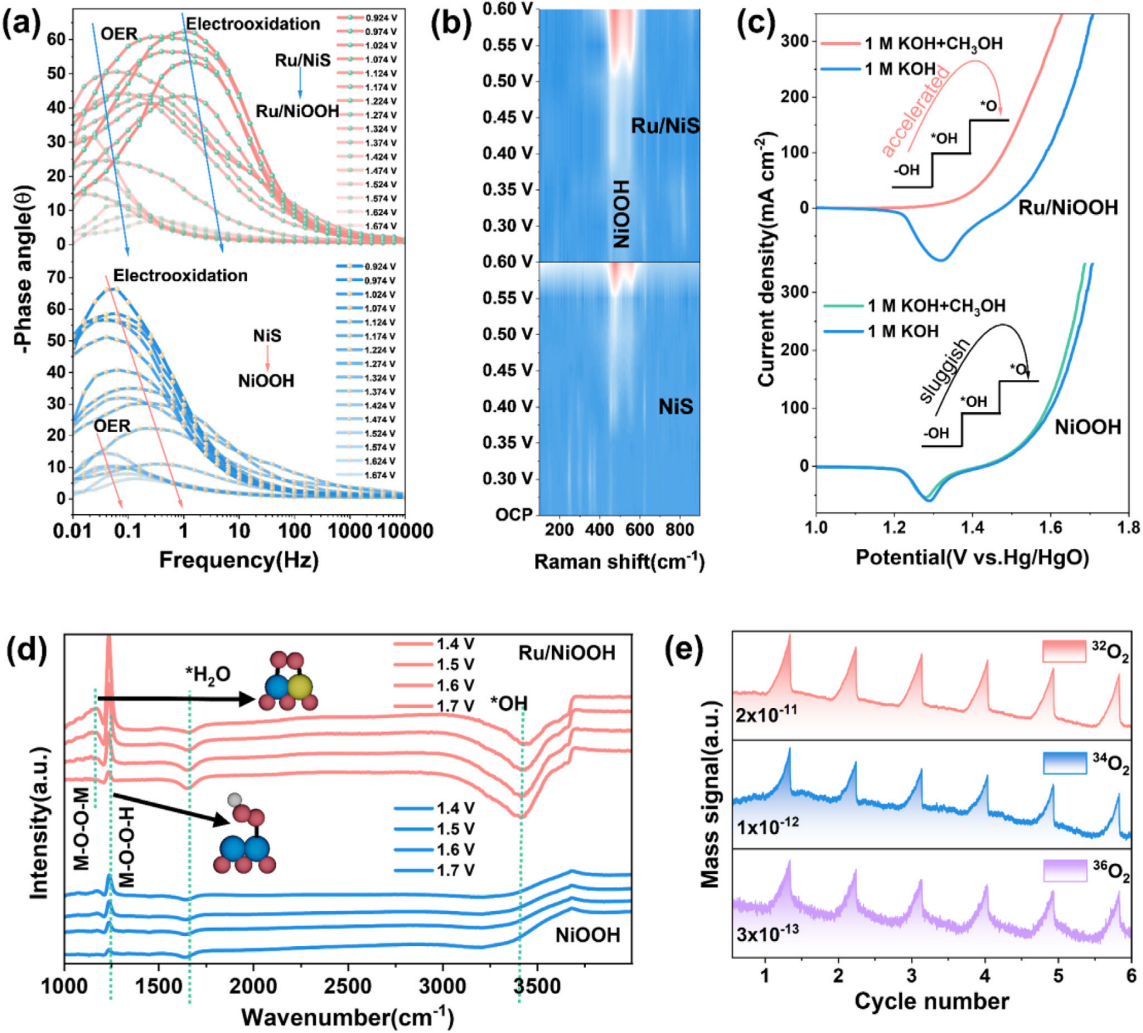
OER kinetics and pathways. (a) In situ EIS, (b) in situ Raman of Ru/NiS and NiS. (c) LSV curves in electrolyte with and without methanol, and (d) in situ FTIR spectra of Ru/NiOOH and NiOOH. (e) DEMS signals of O_2_ products for Ru/NiOOH.

The electrooxidation kinetics were monitored using in situ EIS and in situ Raman spectroscopy. As shown in Figure 5a, the phase angle of Ru/NiS in the high‐frequency region decreases with the potential from 0.924 to 1.374 V vs. RHE, corresponding to the surface reconstruction of Ru/NiS. When the potential exceeds 1.424 V vs. RHE, the phase angle in the high‐frequency region no longer decreases. But it gradually decreases in the low‐frequency region, indicating that the formation of Ru/NiOOH has reached the kinetic limit, triggering OER with a low onset potential. In contrast, there is no significant structural evolution, such as Ru embedding, for the pristine NiS. The OER phase signal only appears after 1.474 V vs. RHE, indicating that the more sluggish OER kinetics of NiS [49]. In situ Raman studies suggest that the reconstruction process begins to generate high‐valence Ni species at 0.40 V vs. Hg/HgO (Figure 5b). The emergence of NiOOH is attributed to the continuous transformation of Ni^2+^ to Ni^3+^ [50]. It is worth noting that Ru/NiS already shows the identical vibration modes of NiOOH near 0.50 V vs. Hg/HgO, while NiS exhibits these signals at a higher potential. Compared to Ru/NiO and Ru/Ni(OH)_2_, Ru/NiS demonstrates a lower reconstruction potential (Figure S14a–c). Except the peaks ascribed to the electrooxidation‐generated NiOOH, instinct peaks at 625 and 695 cm^−1^ are observed in the in situ Raman spectra of Ru/NiS, which are attributed to A_1g_ and B_2g_ vibrations of Ru─O bond (Figure S15a,b). This result proves that the Ru nanoparticles embedding with significant increase of Ru─O bonds at the interface [51].

Methanol (CH_3_OH) molecule is a commonly recognized OPM probe, since it can provide extremely high coverage of *OH and accelerate the oxidation kinetics with *OH participation [34]. Figure 5c shows the polarization curves of Ru/NiS and NiS before and after introducing the CH_3_OH probe into 1 m KOH electrolyte. The results show that the activity of Ru/NiS significantly increases after adding CH_3_OH, indicating that the high abundance of *OH on the surface can promote OER. In contrast, the LSV curve of NiS shows little variation, indicating that the excessive *OH intermediates cannot directly affect OER. This discovery preliminarily confirms that Ru/NiS tends to follow the OPM pathway. CH_3_OH probe significantly accelerates the –OH→*OH→*O reaction steps of Ru/NiS, but has a rare impact on NiS [36].

To confirm the involvement of OPM through reaction intermediates, in situ Fourier transform infrared spectroscopy (FTIR) was carried out. Figure 5d shows the in situ FTIR spectra of Ru/NiS and NIS from 1.4 to 1.7 V vs. RHE. *OH is a common intermediate in the OER process, and its characteristic peak appears at 3450 cm^−1^. The *OH signal of Ru/NiS exhibits a strong adsorption across the entire OER potential gradient, while its signal of NiS appears relatively weak intensity, again clarifying the high surface coverage of *OH on Ru/NiS. In addition, a clear H_2_O peak can be observed at 1650 cm^−1^. What differs is that as the potential increases in the OER region, the peak intensity of Ru/NiS at 1150 cm^−1^ gradually increases, corresponding to the presence of M–O–O–M, which indicates the formation of *O–*O between metal sites in the OPM pathway [38, 39]. Besides, the peak located at 1250 cm^−1^ belongs to the intermediate *OOH in the AEM pathway. These evidences collectively indicates the mechanism switching from AEM toward OPM. Differential electrochemical mass spectrometry (DEMS) analysis was performed on Ru/NiS using isotope labeling (Figure 5e) during OER to ultimately confirm our assumption. Specifically, the DEMS spectra were recorded sequentially using 1 m KOH in H_2_^18^O and H_2_^16^O as the electrolytes. The concentration of ^32^O_2_ (^16^O^16^O) is on the order of 2 × 10^−11^, far exceeding that of ^34^O_2_ (^16^O^18^O, 1 × 10^−12^) and ^36^O_2_ (^18^O^18^O, 1 × 10^−13^). This ^32^O_2_ product mainly arises from H_2_^16^O through the OPM pathway, excluding the occurrence of LOM.

**FIGURE 6:**
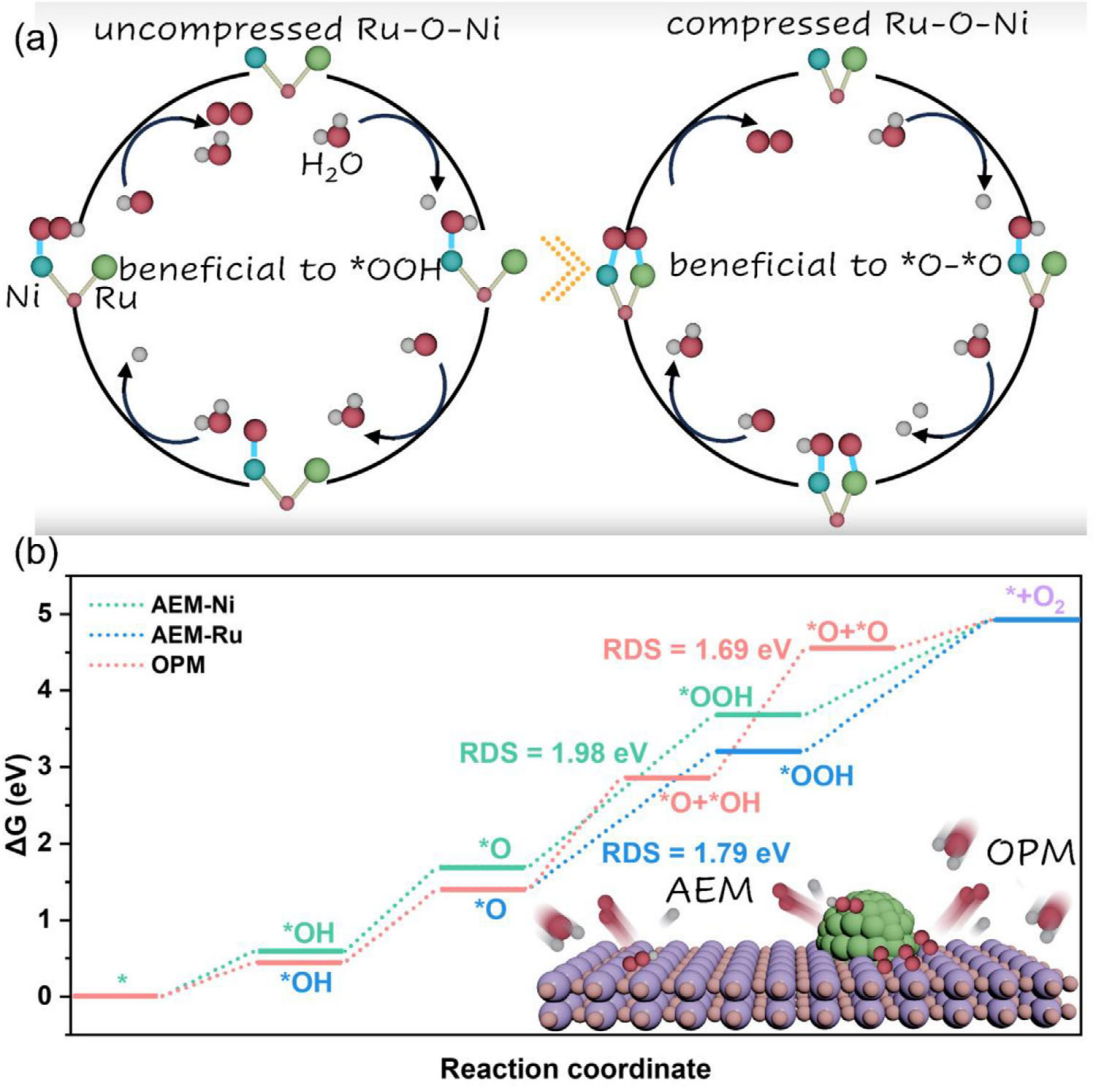
Dual‐path OER mechanism. (a) Schematic diagram of the contribution of the compressed Ru‐O‐Ni interfacial unit to the OPM pathway. (b) Calculated OER free‐energy diagrams at U = 0 V for Ru/NiOOH. An illustration is a schematic diagram of a mechanism transformation.

To reveal the mechanism conversion principle, we compared the roles of uncompressed and compressed Ru─O─Ni unit in the OER process (Figure 6a) and found that the conventionally supported ruthenium nanoparticles on NiOOH are more inclined toward the linear AEM catalytic pathway (*OH→*O→*OOH→*OO). The compressed Ru─O─Ni proposed in this work makes a significant contribution to *O–*O coupling, namely the emergence of OPM. According to the HAADF‐STEM (Figure S16a–d) results, NiOOH (001) is the mainly exposed crystal plane and therefore used for theoretical calculations, where Ru nanoparticles were embedded. First, the electronic structures of NiOOH and Ru/NiOOH were calculated to understand the influence of Ru species on electron redistribution. Compared to the projected density of states (PDOS) of NiOOH [52, 53], as shown in Figure S17a, there was a significant increase in electron aggregation near the E_f_ for Ru/NiOOH, and the electron distribution plots of Ru, O, and Ni highly overlap, indicating the strong interaction of their orbitals and the formation of Ru─O─Ni bonds (Figure S17b). Based on electronic structure regulation, we further explored the possible mechanism on each catalyst. As shown in Figure 6b, the Ni sites on Ru/NiOOH far away from Ru species require an energy of ΔG = 1.98 eV to form *OOH intermediates, following the traditional AEM pathway. At the same time, the Ru sites on the tip of Ru nanoparticles also follow the AEM mechanism, as the formation of *OOH is also the rate determining step (RDS). But its lower energy barrier (ΔG = 1.79 eV) results in enhanced activity. It is noteworthy that, due to the embedding effect, the metal‐support interface brings Ru and Ni bimetallic sites closer, facilitating *O–*O coupling and reducing the corresponding energy barrier of RDS to 1.69 eV and thus triggering the OPM mechanism. This result indicates that the Ru/NiOOH catalyst tends to undergo OPM at the R─O─Ni bridge sites, while in other regions of Ru nanoparticles and NiOOH surface, it operates through the AEM pathway (Figure 6b).

## 3 Discussion

In summary, sufficient experimental evidences confirm that electrooxidation of NiX and embedding of Ru nanoparticles into its surface occur spontaneously, causing local deformation and reducing the distance of Ru─Ni sites. Compared to Ni─Ni sites with larger distances, Ru─Ni bimetallic sites with shorter distances facilitate *O–*O coupling in the OPM pathway, leading to the mechanism switching from AEM to OPM at the metal‐support interface. Ultimately, this reconstructed catalyst demonstrates ultralow overpotential (< 180 mV at 10 mA cm^−2^) and exhibits satisfactory stability. This work establishes adaptive reconstruction‐driven embedding as a universal strategy for activating OER pre‐catalysts, providing a new perspective for developing next‐generation catalysts.

## Author Contributions

**Mingpeng Chen**: conceptualization, writing – review and editing, funding acquisition, resources, methodology. **Feng Liu**: funding acquisition. **Yun Chen**: methodology, data curation. **Huachuan Sun**: funding acquisition. **Yuewen Wu**: conceptualization, writing – original draft, methodology, data curation. **Tong Zhou**: funding acquisition, supervision. **Jin Zhang**: funding acquisition, supervision. **Xinqi Chen**: software, formal analysis. **Qingju Liu**: project administration, writing – review and editing, funding acquisition. **Hao Cui**: funding acquisition. **Dequan Li**: investigation, visualization.

## Conflicts of Interest

There are no conflicts to declare.

## Supporting information




**Supporting File**: advs76026‐sup‐0001‐SuppMat.docx.

## Data Availability

The data that supports the findings of this study are available in the supplementary material of this article.
