# Supplementary material for: Reconstruction of Nickel Chalcogenide Induced Ruthenium Nanoparticles Embedding for Oxygen Evolution: Mechanism Switching Enables Enhanced Catalytic Activity
Source: Adv Sci (Weinh). 2026 Jun 9;13(48):e76026. doi: 10.1002/advs.76026 (PMC13336923; doi:10.1002/advs.76026)
Supplement: Supplementary file 1 [file ADVS-13-e76026-s001.docx]

**Supporting Information**

Reconstruction of nickel chalcogenide induced ruthenium nanoparticles embedding for oxygen evolution: Mechanism switching enables enhanced catalytic activity

Yuewen Wu,^1^ Mingpeng Chen,^1,^ * Xinqi Chen,^1^ Huachuan Sun,^1^ Tong Zhou,^1^ Yun Chen,^1^ Dequan Li,^1^ Jin Zhang,^1^ Feng Liu,^2^ Hao Cui,^2^ and Qingju Liu^1,^ *

^1^ Yunnan Key Laboratory for Micro/Nano Materials & Technology, National Center for International Research on Photoelectric and Energy Materials, School of Materials and Energy, Yunnan University, Kunming 650091, China

^2^ Yunnan Precious Metals Laboratory Co., Ltd., Kunming, 650106, China

* Corresponding authors.

Mingpeng Chen: mpchen@ynu.edu.cn (email)

Qingju Liu: qjliu@ynu.edu.cn (email)

**Synthesis of Ru/NiX**

1.0 mmol ammonium fluoride (NH_4_F), 2.0 mmol urea (CO(NH_2_)_2_), 1.2 mmol sodium thiosulfate/selenium powder (Na_2_S_2_O_3_/Se) and 0.25 mmol nickel nitrate hydrate (Ni(NO_3_)_2_·6H_2_O) were dissolved in 35 mL of deionized (DI) water, and then the pretreated nickel foam (NF, 2 × 3 cm^2^) was immersed in the solution and kept at 135 ℃ for 4 h. NiX (X = S, Se) nanorod arrays were obtained after drying in air. Dissolve 40 mg of ruthenium chloride (RuCl_3_) in 20 mL of deionized water and then soak the prepared Ru/NiS@NF in the solution at room temperature for 48 h, then dry in the air for 6 h.

**Electrochemical measurements**

CHI760e potentiostat was utilized for electrochemical measurements in a conventional three-electrode system. The working electrode, reference electrode, and counter electrode were the as-synthesized sample, Hg/HgO electrode, and carbon rod, respectively. For uniformity, all measurements were calibrated to the RHE using the formula E_RHE_ = E_Hg/HgO_ + 0.098 V + 0.059 × pH. Linear sweep voltammetry (LSV) curves with 95% iR compensation were documented at a scan rate of 2 mV s^−1^. The electrochemical double-layer capacitance (C_dl_) by implementing CV measurements with varying scan rates ranging from 20 to 100 mV/s in the non-faradaic region. The electrochemical impedance spectroscopy (EIS) was performed with a frequency from 100 kHz to 0.01 Hz. DEMS testing method: A two-step experimental method was designed to identify the OER mechanism. Firstly, 20 CV cycles were conducted in a 0.1 M KOH electrolyte prepared using H_2_^18^O as the solvent. If there was a LOM mechanism, ^18^O would enter lattice oxygen vacancies. Subsequently, the catalyst labeled surface was thoroughly cleaned with a large amount of deionized water and purged with nitrogen for 15 minutes. Then, six LSV scans were conducted in a 0.1 M KOH electrolyte prepared using H_2_^16^O as the solvent, and the resulting O_2_ isotope signals were monitored.

**Faraday efficiency**

The Faraday efficiency (FE) is calculated using the following equation:

$$FE=\frac{n*F*V}{Q*V_{m}}\times100\%$$

In the formula, n=4 (OER), F=96485 A·s mol^-1^ (Faraday constant), V_m_=22.4 L/mol (molar volume of a gas), Q=It (total charge), and V is the volume of O_2_ (measured by the drainage method). The current is fixed at 10 mA, and the collected volume of O_2_ gas was recorded every 10 min.

**Materials characterization**

Scanning electron microscopy (SEM, Gemini SEM 460), and transmission electron microscopy (TEM, JEM-2100) were conducted to characterize the morphologies and elements distribution of catalysts. Aberration-corrected high-angle annular dark-field scanning transmission electron microscopy (HAADF-STEM) images were obtained on Thermofisher Spectra 300 equipped with a probe corrector. X-ray diffraction (XRD) data was recorded on an X-ray diffractometer (Rigaku TTR-III) with a Cu Kα source. X-Ray photoelectron spectroscopy (XPS, K-Alpha) tests were conducted using Al source radiation. X-ray absorption spectroscopy (XAS) analysis was carried out at the BL11B beamlines of the Shanghai Synchrotron Radiation Facility located in Shanghai, China, using Si (111) crystal monochromators.

**Theoretical calculations**

All spin-polarized density functional theory (DFT) calculations were performed using the Vienna Ab initio Simulation Package (VASP).[1] The projector augmented wave (PAW) method was employed to describe the interactions between ion cores and valence electrons, [2] while the exchange-correlation interactions were approximated using the Perdew-Burke-Ernzerhof (PBE) functional.[3] he energy cutoff for the plane-wave basis set was set to 500 eV. The convergence criteria for the electronic self-consistent iteration and atomic forces were set to 10^-5^ eV and -0.02 eV/Å, respectively. The Brillouin zone was sampled using a 3×3×1 k-point mesh generated via the Monkhorst-Pack method. [4] The DFT-D3 dispersion correction was included to account for van der Waals interactions. [5] A vacuum layer of 15 Å was applied along the c-axis to prevent spurious interactions between periodic images.

For the structure involving Ru clusters embedded in NIOOH, ab initio molecular dynamics (AIMD) simulations were conducted. The simulations were performed in the NVT ensemble using a Nosé–Hoover thermostat to maintain a constant temperature of 300 K, with a total simulation time of 5 ps. [6]

**Figure S1** XRD patterns of Ru/NiS and NiS. The compositional and structural information of the synthesized pre-catalyst was acquired by using X-ray diffraction (XRD). As shown in Figure S1, the characteristic peaks at 18.5°, 32.3°, 37.4°, 40.5°, and 49.0° are indexed to NiS (PDF #86-2280).

**Figure S2** Morphological characterization of pristine NiS: (a) low and (b) high magnitude SEM images.

**Figure S3** (a) Low and (b) high magnitude SEM images of Ru/NiS.

**Figure S4** (a, b) TEM, (c) HRTEM, (d) SAED image of Ru/NiS.

**Figure S5** High-resolution XPS spectra of O 1s for Ru/NiS before and after OER.

**Figure S6** High-resolution XPS spectra of (a) Ni 2*p* and (b) Ru 3*p* for Ru/NiSe before and after OER. The binding energy of 856.1 eV in Ni 2*p*_3/2_ can be assigned to Ni^2+^ for Ru/NiSe. After electrooxidation, the peak positively shifts by 0.5 eV, closer to the Ni^3+^ state, indicating the appearance of NiOOH on surface.

**Table S1.** Differential Charge Electron Transfer in Ru/NiOOH Model.

| Electron transfer | Species | Electron counts | Average number of electrons |
| --- | --- | --- | --- |
| Negative | Ru | 5.91 | 0.455 |

**Figure S7** Ru *K*-edge EXAFS oscillation functions of Ru/NiS, RuO_2_, and Ru foil.

**Table S2.** The EXAFS fitting parameters at the Ru *K*-edge for Ru/OOH, Ru foil, and RuO_2_.

| Sample | Shell | *CN* | *R*(Å)*^b^* | *σ*^2^(Å^2^)*^c^* | Δ*E*_0_(eV)*^d^* | *R* factor |
| --- | --- | --- | --- | --- | --- | --- |
| Ru foil | Ru-Ru | 6* | 2.24 | 0.63 | 7.96 | 0.014545 |
| RuO_2_ | Ru-O | 3* | 1.53 | 0.89 | 8.36 | 0.007701 |
|  | Ru-Ru | 3* | 2.23 | 0.90 | 10.23 |  |
| Ru/OOH | Ru-O | 2.7 | 1.51 | 0.95 | 10.38 | 0.008526 |
|  | Ru-Ru | 3.1 | 2.23 | 0.73 | 4.77 |  |

*CN*, coordination number; *^b^R*, distance between absorber and backscatter atoms; *^c^σ*^2^, Debye-Waller factor to account for both thermal and structural disorders; *^d^ΔE*_0_, inner potential correction; *R* factor indicates the goodness of the fit. S_0_^2^ was fixed to 0.70. A reasonable range of EXAFS fitting parameters: 0.600 < *Ѕ*_0_^2^ < 1.000; *CN >* 0; *σ*^2^ > 0 Å^2^; |Δ*E*_0_| < 10 eV; *R* factor < 0.02.

**Figure S8** LSV polarization curve of samples soaked in 0.5, 1.0, 1.5, 2.0, 2.5 mg/mL concentration.

**Figure S9** (a-c) CV curves of Ru/NiX, NiX and RuO_2_ from 20 mV/s to 100 mV/s scan rates. (d) Linear fitting of the capacitive currents against CV scan rates for Ru/NiX, NiX and RuO_2_.

**Figure S10** Normalized LSV curves of as-prepared catalysts.

**Figure S11** Faradaic efficiency of Ru/NiS for OER.

**Figure S12** Chronopotentiometric test of Ru/NiS at a current density of 250 mA cm^-2^.

**Figure S13** (a-c) SEM images, (d-f) TEM images, (g-h) HAADF-STEM images and (i) Elements mapping of Ru/NiS after OER.

**Table S3** Fitting results of Nyquist plots on Ru/NiX, NiX, RuO_2_ obtained from Figure S9b.

| **Catalyst** | **Rs (Ω)** | **Rct (Ω)** | **CPE-T** | **CPE-P** |
| --- | --- | --- | --- | --- |
| Ru/NiS | 1.37 Ω | 0.94 Ω | 1.878 | 0.545 |
| NiS | 1.40 Ω | 9.80 Ω | 1.085 | 0.697 |
| RuO_2_ | 1.39 Ω | 29.63 Ω | 0.770 | 0.791 |
| Ru/NiSe | 1.35 Ω | 1.81 Ω | 1.625 | 0.577 |
| NiSe | 1.41 Ω | 31.84 Ω | 1.091 | 0.897 |

**Table S4.** Comparison of the OER performance with recently reported electrocatalysts tested in alkaline media.

| **Catalyst** | **η_50_(mV)** | **η_100_(mV)** | **Ref.** |
| --- | --- | --- | --- |
| Ru/NiS | 220 | 267 | This work |
| Ru@Cr-FeMOF | 230 | 260 | [7] |
| Ir_SA_-Ni_2_P | 210 | 270 | [8] |
| Ru-VS_2_@CC | 245 | 280 | [9] |
| Ru_9.1_-NiFe-MOF/NFF | 250 | 288 | [10] |
| FeOOH@NiFe LDH | 255 | 290 | [11] |
| RuFe@NF | 265 | 290 | [12] |
| P-doped Rh SAC-Co_3_O_4_/NF | 268 | 290 | [13] |
| Ru/Rh-FeOOH@Ti_3_C_2_T_x_ | 290 | 320 | [14] |
| Ni_0.85_Se-O/CN | 295 | 330 | [15] |
| CoVRu LDH | 296 | 325 | [16] |
| Ru/NiFe(OH)_x_/NiFe-MOF | 320 | 345 | [17] |
| Ru, Ni–CoP | 322 | 350 | [18] |
| SARu/NiFe LDH | 326 | 345 | [19] |
| RuO_2_/CoO_x_ | 345 | 370 | [20] |
| Ru/Co_3_O_4–x_ | 350 | 377 | [21] |
| (Ru-Co)O_x_-350 | 351 | 374 | [22] |
| Ru/B–Ni_2_P/Ni_5_P_4_ | 355 | 379 | [23] |
| Co_0.89_Fe_0.11_O-N | 360 | 391 | [24] |
| Ru-FeRu@C/NC | 410 | 451 | [25] |
| Ru-H_2_O/CC-350 | 430 | 458 | [26] |

**Table S5** ICP analysis of electrolyte after OER reaction.

| element | Concentration (ug/L) |
| --- | --- |
| Ru | 568.6 |
| Ni | 323.1 |

**Figure S14** In-situ Raman studies (a) Ru/NiO, (b) Ru/Ni(OH)_2_ and (c) Ru/NiOOH.

**Figure S15** (a) Raman spectrum of RuO_2_ and (b) in-situ Raman spectra of Ru/NiS during alkaline OER.

**Figure S16** (a) TEM image and (b-d) HRTEM images of Ru/NiOOH.

**Figure S17** PDOS of (a) NiOOH and (b) Ru/NiOOH.

**References**

[1]	G. Kresse, J. Hafner, *Physical Review B* **1993**, *48* (17), 13115, <https://doi.org/10.1103/PhysRevB.48.13115>.

[2]	P. E. Blöchl, *Physical Review B* **1994**, *50* (24), 17953, <https://doi.org/10.1103/PhysRevB.50.17953>.

[3]	J. P. Perdew, J. A. Chevary, S. H. Vosko, K. A. Jackson, M. R. Pederson, D. J. Singh, C. Fiolhais, *Physical Review B* **1992**, *46* (11), 6671, <https://doi.org/10.1103/PhysRevB.46.6671>.

[4]	H. J. Monkhorst, J. D. Pack, *Physical Review B* **1976**, *13* (12), 5188, <https://doi.org/10.1103/PhysRevB.13.5188>.

[5]	S. Grimme, *Journal of Computational Chemistry* **2006**, *27* (15), 1787, <https://doi.org/https://doi.org/10.1002/jcc.20495>.

[6]	G. J. Martyna, M. L. Klein, M. Tuckerman, *J. Chem. Phys.* **1992**, *97* (4), 2635, <https://doi.org/10.1063/1.463940> %J The Journal of Chemical Physics.

[7]	C. Zhao, J. Wang, Y. Gao, J. Zhang, C. Huang, Q. Shi, S. Mu, Q. Xiao, S. Huo, Z. Xia, J. Zhang, X. Lu, Y. Zhao, *Advanced Functional Materials* **2024**, *34* (7), 2307917, <https://doi.org/https://doi.org/10.1002/adfm.202307917>.

[8]	Q. Wang, Z. Zhang, C. Cai, M. Wang, Z. L. Zhao, M. Li, X. Huang, S. Han, H. Zhou, Z. Feng, L. Li, J. Li, H. Xu, J. S. Francisco, M. Gu, *Journal of the American Chemical Society* **2021**, *143* (34), 13605, <https://doi.org/10.1021/jacs.1c04682>.

[9]	Z. Hou, C. Cui, Y. Yang, T. Zhang, *Small* **2023**, *19* (29), 2207170, <https://doi.org/https://doi.org/10.1002/smll.202207170>.

[10]	W. Jiang, J. Wang, Y. Jiang, Y. Wu, B. Liu, X. Chu, C. Liu, G. Che, Y. Lu, *Journal of Materials Chemistry A* **2023**, *11* (6), 2769, <https://doi.org/10.1039/D2TA06560F>.

[11]	M. Zhang, B. Wang, H. Sun, M. Chen, T. Zhou, D. Li, B. Xiao, J. Zhao, Y. Zhang, J. Zhang, Q. Liu, *International Journal of Hydrogen Energy* **2024**, *60*, 1215, <https://doi.org/https://doi.org/10.1016/j.ijhydene.2024.02.234>.

[12]	H. Liu, Q. Jia, S. Huang, L. Yang, S. Wang, L. Zheng, D. Cao, *Journal of Materials Chemistry A* **2022**, *10* (9), 4817, <https://doi.org/10.1039/D1TA10546A>.

[13]	Y. Gu, X. Wang, A. Bao, L. Dong, X. Zhang, H. Pan, W. Cui, X. Qi, *Nano Research* **2022**, *15* (10), 9511, <https://doi.org/10.1007/s12274-022-4738-z>.

[14]	B. Zhang, J. Shan, X. Wang, Y. Hu, Y. J. S. Li, **2022**, e2200173.

[15]	C. Zhang, W. Xu, S. Li, X. Wang, Z. Guan, M. Zhang, J. Wu, X. Ma, M. Wu, Y. Qi, *Chemical Engineering Journal* **2023**, *454*, 140291, <https://doi.org/https://doi.org/10.1016/j.cej.2022.140291>.

[16]	K. Zeng, M. Tian, X. Chen, J. Zhang, M. H. Rummeli, P. Strasser, J. Sun, R. Yang, *Chemical Engineering Journal* **2023**, *452*, 139151, <https://doi.org/https://doi.org/10.1016/j.cej.2022.139151>.

[17]	D. Liu, H. Xu, C. Wang, C. Ye, R. Yu, Y. Du, *Journal of Materials Chemistry A* **2021**, *9* (43), 24670, <https://doi.org/10.1039/D1TA06438J>.

[18]	Y. Song, J. Cheng, J. Liu, Q. Ye, X. Gao, J. Lu, Y. Cheng, *Applied Catalysis B: Environmental* **2021**, *298*, 120488, <https://doi.org/https://doi.org/10.1016/j.apcatb.2021.120488>.

[19]	Y. Yang, Q.-N. Yang, Y.-B. Yang, P.-F. Guo, W.-X. Feng, Y. Jia, K. Wang, W.-T. Wang, Z.-H. He, Z.-T. Liu, *ACS Catalysis* **2023**, *13* (4), 2771, <https://doi.org/10.1021/acscatal.2c05624>.

[20]	K. Du, L. Zhang, J. Shan, J. Guo, J. Mao, C.-C. Yang, C.-H. Wang, Z. Hu, T. Ling, *Nature Communications* **2022**, *13* (1), 5448, <https://doi.org/10.1038/s41467-022-33150-x>.

[21]	C.-Z. Yuan, S. Wang, K. San Hui, K. Wang, J. Li, H. Gao, C. Zha, X. Zhang, D. A. Dinh, X.-L. Wu, Z. Tang, J. Wan, Z. Shao, K. N. Hui, *ACS Catalysis* **2023**, *13* (4), 2462, <https://doi.org/10.1021/acscatal.2c04946>.

[22]	C. Wang, H. Shang, J. Li, Y. Wang, H. Xu, C. Wang, J. Guo, Y. Du, *Chemical Engineering Journal* **2021**, *420*, 129805, <https://doi.org/https://doi.org/10.1016/j.cej.2021.129805>.

[23]	Y. Wang, Q. Sun, Z. Wang, W. Xiao, Y. Fu, T. Ma, Z. Wu, L. Wang, *Journal of Materials Chemistry A* **2022**, *10* (30), 16236, <https://doi.org/10.1039/D2TA02685F>.

[24]	Q. Du, P. Su, Z. Cao, J. Yang, C. A. H. Price, J. Liu, *Sustainable Materials and Technologies* **2021**, *29*, e00293, <https://doi.org/https://doi.org/10.1016/j.susmat.2021.e00293>.

[25]	W. Feng, Y. Feng, J. Chen, H. Wang, Y. Hu, T. Luo, C. Yuan, L. Cao, L. Feng, J. Huang, *Chemical Engineering Journal* **2022**, *437*, 135456, <https://doi.org/https://doi.org/10.1016/j.cej.2022.135456>.

[26]	M. You, X. Du, X. Hou, Z. Wang, Y. Zhou, H. Ji, L. Zhang, Z. Zhang, S. Yi, D. Chen, *Applied Catalysis B: Environmental* **2022**, *317*, 121729, <https://doi.org/https://doi.org/10.1016/j.apcatb.2022.121729>.
